# Negative problem orientation and social problem solving in esports: indirect role of cognitive flexibility

**DOI:** 10.3389/fpsyg.2026.1797082

**Published:** 2026-06-18

**Authors:** Alp Kaan Kilci, Seyhan Bekir, Serhat Yalciner, Nahit Ozdayi

**Affiliations:** 1 Faculty of Sport Sciences, Sport Management Department, Balıkesir University, Balıkesir, Türkiye; 2 Directorate of Sports Sciences Application and Research Center, Balikesir University, Balıkesir, Türkiye; 3 Faculty of Science and Letters, Department of Psychology, Demiroglu Bilim University, Istanbul, Türkiye; 4 Faculty of Sport Sciences, Coaching Education Department, Balıkesir University, Balıkesir, Türkiye

**Keywords:** cognitive flexibility, esports, mediator, negative problem orientation, social problem-solving

## Abstract

**Background:**

Mental process is a cornerstone of success in the rapid sports industry, where players must navigate high-pressure, dynamic environments. Cognitive flexibility is essential for adaptability and effective problem-solving, yet its relationship with negative psychological traits remains underexplored. This study investigates the mediating role of cognitive flexibility in the relationship between negative problem orientation and social problem-solving competence among esports players.

**Methods:**

The study sample consisted of 966 esports players from Türkiye, categorized as semi-professionals, professionals, and hardcore players. Data were collected using the Negative Problem Orientation Questionnaire, the Social Problem-Solving Skills Inventory, and the Cognitive Flexibility Scale. Statistical analysis was conducted using the SPSS PROCESS macro (Model 4), employing bootstrapping methods to evaluate the significance of direct and indirect effects within the mediation model.

**Results:**

Mediation analysis revealed that cognitive flexibility significantly mediates the relationship between negative problem orientation and social problem-solving competence (indirect effect = 0.97, 95% CI [0.4805 1.485]). Direct effects were also significant: negative problem orientation predicted social problem-solving, while cognitive flexibility showed a strong positive impact on problem-solving competence.

**Conclusion:**

Cognitive flexibility serves as a vital psychological mechanism that may reduce the adverse influence of negative problem orientation and enhance social problem-solving competence in esports players. These findings suggest that interventions designed to strengthen cognitive flexibility such as scenario-based decision-making drills, mindfulness-based attention training, and stress-regulation exercises may contribute to better adaptability, psychological resilience, and problem-solving performance in competitive esports contexts.

## 1 Introduction

Esports is a rapidly growing competitive field within the digital gaming world, encompassing a broad spectrum of players with different levels of experience and expertise. Success in this domain depends not only on technical or motor skills but also on mental competence, cognitive regulation, and adaptive problem-solving. Competitive video games require a wide range of cognitive skills, including perception, attention, memory, planning, and decision-making. The constant need for problem-solving, strategy development, and rapid decision-making during gameplay highlights the cognitive demands of esports and the potential contribution of gaming experiences to cognitive development (Bediou et al., 2018, 2023; Sauce et al., 2022). As esports players’ levels of experience increase, differences in cognitive abilities may become more pronounced. Compared with less experienced players, elite esports players have been reported to show advantages in several cognitive skills, particularly spatial working memory, mental rotation, and task switching (Bediou et al., 2018; Kang et al., 2020; Tanaka et al., 2013; Li et al., 2020).

Esports environments are characterized by continuous real-time information updating, high-density perceptual input, compressed reaction windows, and digitally mediated team communication. Competitive performance depends not merely on motor execution but also on the rapid integration of visual tracking, strategic anticipation, multitasking, and adaptive decision-making under uncertainty (Himmelstein et al., 2017; McNulty et al., 2023; Campbell et al., 2018; Powers et al., 2013; Vaamonde et al., 2018). Players must inhibit impulsive responses, shift flexibly between tactical goals, and continuously update working memory representations in response to dynamic in-game feedback, thereby engaging executive functions at a sustained and intensified level (Castel et al., 2005; Toth et al., 2021). Moreover, real-time voice-based coordination within teams introduces a substantial social-cognitive load, further increasing demands on attentional control, cognitive flexibility, and executive regulation (Maier, 2024; Eccles and Tenenbaum et al., 2004). Therefore, esports can be considered a digitally mediated performance domain in which executive functions operate as central regulatory mechanisms governing competitive success.

Empirical evidence also indicates that esports performance is associated with reaction time efficiency, attentional allocation under load, and adaptive regulation during competitive stress. Experimental and field-based findings suggest that competitive gaming elicits measurable cognitive and physiological activation patterns, with performance variability linked to response execution speed and attentional control capacity (Sousa et al., 2020; Pedraza-Ramirez et al., 2025). Stress exposure in professional esports has also been shown to influence in-game decision processes and behavioral regulation, emphasizing the functional relevance of executive control mechanisms for competitive outcomes (Leis et al., 2022). In team-based contexts, performance additionally depends on coordinated communication and synchronized tactical adaptation, as disruptions in team processes and communication flow may impair collective efficiency (Gisbert-Pérez et al., 2024; Freeman and Wohn, 2019). Within this performance-driven framework, cognitive flexibility should not be treated merely as a general cognitive trait applied to esports, but as a central executive mechanism that links cognitive regulation to dynamic, real-time competitive performance.

### 1.1 Cognitive flexibility: definition and strategic importance in Esports

Cognitive flexibility is frequently discussed in psychological and sports literature as an important theoretical and practical construct (Başoğlu et al., 2025; Mazzoccante et al., 2019; Korucuk et al., 2025; Zhang et al., 2026). It was defined by Spiro and Jehng (2012) as the ability to restructure knowledge in response to changing situational demands. In a broader sense, cognitive flexibility refers to the ability to adapt to changing conditions, switch between ideas, modify ineffective strategies, and approach problems from multiple perspectives. It has also been described as a form of fluid intelligence that enables individuals to generate different solutions to various situations (Silver et al., 2004) and to modify their performance according to contextual demands (Stevens, 2009). Individuals with higher cognitive flexibility are more capable of shifting between different contexts and adapting their responses, whereas those with lower cognitive flexibility may experience greater difficulty in managing changing situations (Cox, 1980).

In esports, cognitive flexibility has strategic importance because players are constantly required to respond to uncertain, dynamic, and rapidly changing conditions. During gameplay, players may need to revise an unsuccessful strategy, reinterpret opponents’ actions, adapt to teammates’ decisions, or shift between individual and team-based tactical goals within very short time intervals. Cognitive flexibility therefore supports goal-directed behavior, reasoning, planning, and problem-solving in competitive environments. Previous studies have also shown that individuals with lower cognitive flexibility may encounter difficulties in self-efficacy, preparedness, attention, and situation awareness, whereas individuals with higher cognitive flexibility tend to display these skills more successfully (Martin and Rubin, 1995; Martin and Anderson, 1996, 1998). In this respect, cognitive flexibility represents a key executive function that may help esports players adapt to changing game demands and maintain effective performance under pressure.

### 1.2 Social problem solving: esports, team dynamics, and communication

Social problem solving is another important construct in the esports context. It refers to the cognitive, emotional, and behavioral strategies individuals use to address social challenges and is defined as the application of problem-solving processes to social contexts (D’Zurilla et al., 1998; D’Zurilla and Nezu, 1982). While general problem-solving includes the cognitive and behavioral processes used to deal with different types of problems, social problem-solving represents the interpersonal dimension of this ability within relationships and social interactions. It contributes not only to individuals’ capacity to resolve problems but also to the formation of quality relationships and improved quality of life (Felce and Perry, 1995; Wallander et al., 2001).

Although social problem-solving has traditionally been conceptualized within everyday interpersonal contexts, gaming environments have also been recognized as structured spaces in which such processes can be modeled and refined (Klabbers, 2014). More broadly, social problem-solving reflects a collective decision-making process through which social actors interpret shared demands and pursue short- and long-term goals (Schön, 1983; Patašienė et al., 2014). In esports, this process becomes more accelerated and performance contingent. Team-based esports require players to communicate tactical information, respond to teammates’ decisions, manage interpersonal tension, and coordinate collective strategies under time pressure. Therefore, social problem-solving competence may contribute to both individual and team performance by supporting communication quality, adaptive decision-making, and coordinated action.

The distinction between general social problem-solving and esports-related decision-making lies not in their social nature but in the degree of executive regulation required. Esports players must solve interpersonal and tactical problems while simultaneously managing fast-changing visual information, emotional pressure, and team communication. Thus, social problem-solving in esports is closely related to executive functions, particularly cognitive flexibility. Cognitive flexibility may enable players to process new information, consider alternative solutions, and adapt their decisions to changing social and competitive demands (Taş and Deniz, 2018). Individuals who can generate multiple alternatives to a problem are likely to demonstrate more adaptive responses than individuals who rely on a single rigid strategy (Spivack et al., 1976). Within this framework, cognitive flexibility may help socially oriented problem-solving tendencies translate into adaptive in-game decision-making.

### 1.3 Negative problem orientation: attitude as an obstacle to performance

However, cognitive and social problem-solving processes are not determined only by technical skill or cognitive capacity; they are also influenced by individuals’ attitudes toward problems. Attitudes are tendencies that shape thoughts, feelings, and behaviors toward an object, person, or event (Kağıtçıbaşı, 1999), and they include affective, cognitive, and behavioral components (Han and Carpenter, 2014). An individual’s attitude toward a particular situation may directly influence behavior in that area (Holzman, 2010; Üstüner, 2006). In this regard, negative problem orientation represents a dysfunctional attitude toward problems and may constitute an important psychological barrier to effective problem-solving.

Negative problem orientation is characterized by perceiving problems as threats, doubting one’s own problem-solving ability, expecting negative outcomes, and experiencing pessimistic thoughts during difficult situations (Davey, 1994; Dugas et al., 1995). Such an orientation may hinder problem-solving skills because individuals may focus more on threat, failure, or inadequacy than on generating effective solutions. In competitive esports, where players are continuously required to react quickly, regulate emotions, and develop strategies, negative problem orientation may be particularly disadvantageous. Players with high negative problem orientation may experience anxiety, stress, concentration problems, and decision-making difficulties during competition. These difficulties may weaken both individual adaptability and team-based problem-solving efficiency.

In contrast, a more constructive attitude toward problems may support confidence in one’s abilities and facilitate effective problem-solving behavior (Gosselin et al., 2005). Therefore, reducing the restrictive influence of negative problem orientation may be important for enhancing players’ adaptability, communication, and cohesion in dynamic esports environments. The critical issue is not only whether players possess problem-solving skills, but whether they can access and use these skills effectively under pressure. Negative problem orientation may create a psychological blockage that prevents players from evaluating situations calmly, generating alternatives, and communicating effectively with teammates.

### 1.4 Cognitive flexibility as a mediation mechanism

Cognitive flexibility may explain how this blockage is reduced. When players perceive problems as threats, they may become more rigid in their interpretations and less capable of producing alternative responses. Cognitive flexibility, however, may help players reinterpret problems, shift perspectives, and generate more adaptive solutions. In other words, flexible players may be less likely to remain trapped in negative evaluations and more likely to transform difficult situations into manageable problem-solving tasks. This mechanism is especially relevant in esports, where rapid adaptation and effective communication are required during constantly changing competitive conditions.

Accordingly, cognitive flexibility may function as a mediating mechanism in the relationship between negative problem orientation and social problem-solving competence. Negative problem orientation may restrict adaptive thinking by increasing pessimistic expectations, emotional overload, and rigid interpretations of problems. Cognitive flexibility may reduce these restrictive effects by enabling players to consider alternative explanations, regulate their responses, and approach social or tactical problems more constructively. Therefore, cognitive flexibility may serve as the executive-regulatory process through which negative problem orientation is linked to social problem-solving competence.

### 1.5 Aim, unique value of the study and hypotheses

The present study aims to examine the relationships among negative problem orientation, cognitive flexibility, and social problem-solving competence among esports players, with particular emphasis on the mediating role of cognitive flexibility. Although previous studies have emphasized the cognitive demands of esports, limited attention has been given to how negative attitudes toward problems may influence social problem-solving competence through executive-function mechanisms.

The unique value of this study lies in positioning cognitive flexibility as a regulatory mechanism that may explain how negative problem orientation is associated with social problem-solving competence in esports. Rather than treating cognitive flexibility only as a general cognitive skill, the present study conceptualizes it as a psychological process that may help players overcome rigid, threat-based evaluations of problems and respond more adaptively to social and competitive demands. Based on this theoretical framework, the following hypotheses were developed:

*H1*: There is a significant relationship among social problem-solving competence, negative problem orientation, and cognitive flexibility.

**Figure 1:**
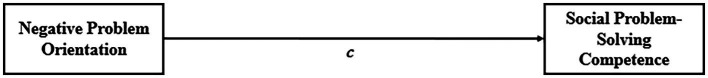
Total effects of negative problem orientation social problem-solving in esport.

*H2*: Negative problem orientation significantly predicts social problem-solving competence (Figure 1).

**Figure 2:**
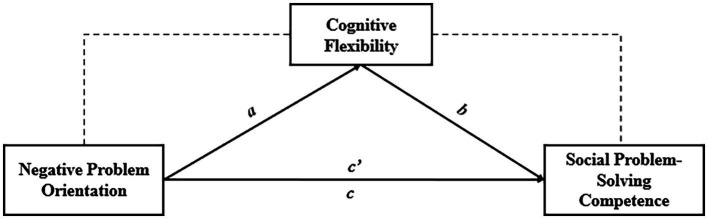
The mediation model proposes that negative problem orientation indirectly predicts social problem-solving through cognitive flexibility.

*H3*: Cognitive flexibility mediates roles in the relationship between negative problem orientation and social problem-solving competence (Figure 2).

## 2 Materials and methods

### 2.1 Research design and participants

The research is based on a comprehensive methodological framework: descriptive and relational analysis combined to shed light on the dependent and independent variable status quo and their interactive analysis. The primary purpose of utilizing these models is to shed light on the relationship between negative problem orientation and social problem-solving within the context of esports. Additionally, descriptive and relational models have been applied to conduct an in-depth analysis of the mediating role of cognitive flexibility in this relationship. The hypotheses central to our research framework form the core assumptions of the study.

Research hypotheses aim to explain how negative problem orientation affects social problem-solving skills both directly and indirectly within the context of esports, while elucidating the mediating role of cognitive flexibility in this relationship. In this regard, cognitive flexibility is considered a mediating variable in the relationship between negative problem orientation and social problem-solving capacity. The role of cognitive flexibility in this context has been examined through a comprehensive analysis to determine whether it enables individuals to reframe their negative problem orientation and develop more effective social problem-solving strategies. This mediating role is regarded as a critical element in understanding the impact of negative problem orientation on social problem-solving.

Semi-professionals, professionals, hardcore players and players who have participated in at least one tournament in the field of esports in organizations operating in the field of esports in Türkiye were included in this study. The criteria for sampling method was applied in the study. According to McKay et al. (2022), esports players can be classified as Tier 3 and Tier 2 players who compete at the national level, and Toth et al. (2021) classify them as “hardcore” players and players. This study focuses on individuals in Türkiye who are actively engaged in esports gaming. The age distribution of the participants reveals that 26.1% are between 18 and 20 years old, 38.6% are between 21 and 23 years old, 17.8% are between 24 and 26 years old, and 17.5% are 27 years or older. Regarding esports experience, 38.5% of the participants have 0–1 year of experience, 16.8% have 2–3 years, 12.3% have 4–5 years, and 32.4% have more than 6 years of experience. With a total of 966 participants, this study provides significant insights into the age and experience levels of esports players.

### 2.2 Data collection tools

In this study, the Negative Problem Orientation Questionnaire, the Social Problem-Solving Inventory and Cognitive Flexibility were used as data collection tools.

#### 2.2.1 Negative problem orientation questionnaire (NPOQ)

The original form was developed by Gosselin et al. (2005) and adapted to Turkish by Akyay (2016). This self-report questionnaire is designed to measure perceived inadequacies of individuals in dealing with day-to-day problems. The internal consistency Cronbach’s Alpha coefficient of this inventory was 0.87, and it has been structured as a Likert-type scale with 12 items, response options ranging from 1 to 5 (from Strongly Disagree to Strongly Agree), giving a total score that ranges between 12 and 60. The scale is of a unidimensional structure, and no subscales. The scale score is the total response to all items. Higher scores reflect a greater negative orientation toward problems. The Cronbach’s Alpha coefficient of internal consistency reliability was given as 0.906 in the scale adapted to Turkish by Akyay (2016).

#### 2.2.2 Social problem-solving inventory – short form (SPSI-SF)

Social Problem-Solving Inventory was originally developed by D’Zurilla and Chang (1995). The Inventory was revised and refined by Maydeu-Olivares and D’Zurilla (1996). In its final version, named the Revised Social Problem-Solving Inventory (SPSI-R), it was renamed by D’Zurilla et al. (2002). The scale was adapted into Turkish by Çekici (2009). To assess its reliability, the scale underwent internal consistency and test–retest reliability analyses. Unlike some other scales, the SPSI-R does not include reverse-scored items. The total score ranges from 0 to 100, with higher scores indicating a higher level of social problem-solving ability and lower scores reflecting poorer problem-solving proficiency. This scoring system enables the evaluation of individuals’ capacity to effectively approach and resolve social challenges.

#### 2.2.3 Cognitive flexibility scale

Cognitive Flexibility Scale is used to measure participants’ levels of cognitive flexibility which developed by Martin and Rubin (1995) and adapted into Turkish by Çelikkaleli (2014). The scale consists of 12 items structured under a single dimension. The validity and reliability analyses conducted during the Turkish adaptation revealed an internal consistency coefficient of 0.74. Additionally, the test–retest correlation coefficient was reported as 0.98, and the split-half reliability coefficient was determined to be 0.77.

### 2.3 Ethical approval, procedure and statistics analysis

This study was conducted in accordance with the principles of the Declaration of Helsinki. Ethical approval for the research was granted by the Ethics Committee for Social and Human Sciences Research at Balıkesir University on June 27, 2024 (Protocol No: 2024/6). Participants were recruited under the guidance of the researchers and through digital platforms. Specifically, online surveys were distributed to connect with individuals engaged in esports gaming. To ensure participants fully understood the objectives of the study, communication was established with online communities and platforms frequented by esports players. The primary aims of the study were explained in detail. Following this explanation, players were invited to participate voluntarily. It was emphasized that participation was entirely optional and that participants had the right to withdraw from the study at any time during the data collection process if they experienced discomfort or hesitation. Each survey administered to the participants included standardized instructions carefully designed to emphasize the importance of maintaining the confidentiality of their responses. Participants were encouraged to provide honest and sincere answers, fostering an environment of transparency and accuracy that contributes to the integrity of the research. In this study, the PROCESS macro for mediation analysis developed by Hayes (2018) was used to analyse indirect effects and their statistical significance using bootstrapping. According to Hayes’s method, the mediated role of a variable is established if the indirect effects from the independent variable on the dependent variable through the mediator is statistically significant. Bootstrapping methods are used to test this by calculating the confidence interval of the indirect effect (Hayes, 2022). A significant indirect effect was found, indicating that cognitive agility is a mediating factor in the relationship between pessimism and the ability to solve social problems.

In the study the relationships among social problem-solving, negative problem orientation, and cognitive flexibility were examined. The data were analyzed using SPSS PROCESS (Model 4) version. Before conducting the analysis, the data were checked for linearity and multicollinearity issues. Out of the 966 data points, no outliers disrupting the analysis were detected and all data points were included in the analysis. Data were collected online through Google Forms on a voluntary basis.

## 3 Results

The results of these checks showed no multicollinearity issues. Table 1 presents the descriptive statistics, including the number of participants (N), means, standard deviations (SD), variance inflation factors (VIF), and confidence intervals (CI). Additionally, the correlation matrix was calculated to show the relationships among the variables.

**Table 1:** Descriptive statistics, linearity, normality, multicollinearity, and correlations.

Variables	*N*	Means	SD	VIF	CI	1	2	3
Social problem-solving competence (1)	966	2.87	0.66		1.00	1		
Negative problem orientation (2)	966	1.68	0.77	4.81	1.01	−0.17^**^	1	
Cognitive flexibility (3)	966	3.35	0.99	10.74	1.01	0.63^**^	0.10^**^	1

The symbol ** indicates statistical significance at **p < 0.01.

As shown in Table 2, social problem-solving competence is negatively correlated with negative problem orientation (*r* = −0.17, *p* < 0.01) and cognitive flexibility (*r* = 0.63, *p* < 0.01). Similarly. Negative problem orientation is positively correlated with cognitive flexibility (*r* = 0.10, *p* < 0.01). Table 2 shows that social problem-solving competence significantly predicts cognitive flexibility (*β* = −2.289, *p* < 0.001).

**Table 2:** The result of the regression analysis.

Predictor	*β*	*SE*	*p*	*F*	*R*	*R^2^*
Constant	60,289	0.79	<0.00	28,21	0.17	0.02
Negative problem orientation	−2.289	0.43	<0.01			

The results regarding the mediating role of cognitive flexibility in the relationship between negative problem orientation and social problem-solving competence are shown in Table 3.

**Table 3:** Mediational model coefficients.

Predictors	Cognitive flexibility			Social problem-solving competence		
		*b*	*p*		*b*	*p*
Negative Problem Orientation	*a*	0.10	0.00	*c′*	−3.26	0.00
Cognitive Flexibility		------	------	*b*	8.92	0.00
Constant	*i*_1_	3,24	0.00	*i_2_*	31.33	0.00
	*R* = 0.10. *R*^2^ = 0.01			*R* = 0.68. *R*^2^ = 0.46		
	*F*_(1. 963)_ = 52.17, *p* < 0.001			*F*_(2. 963)_ = 419.50, *p* < 0.001		

**Figure 3:**
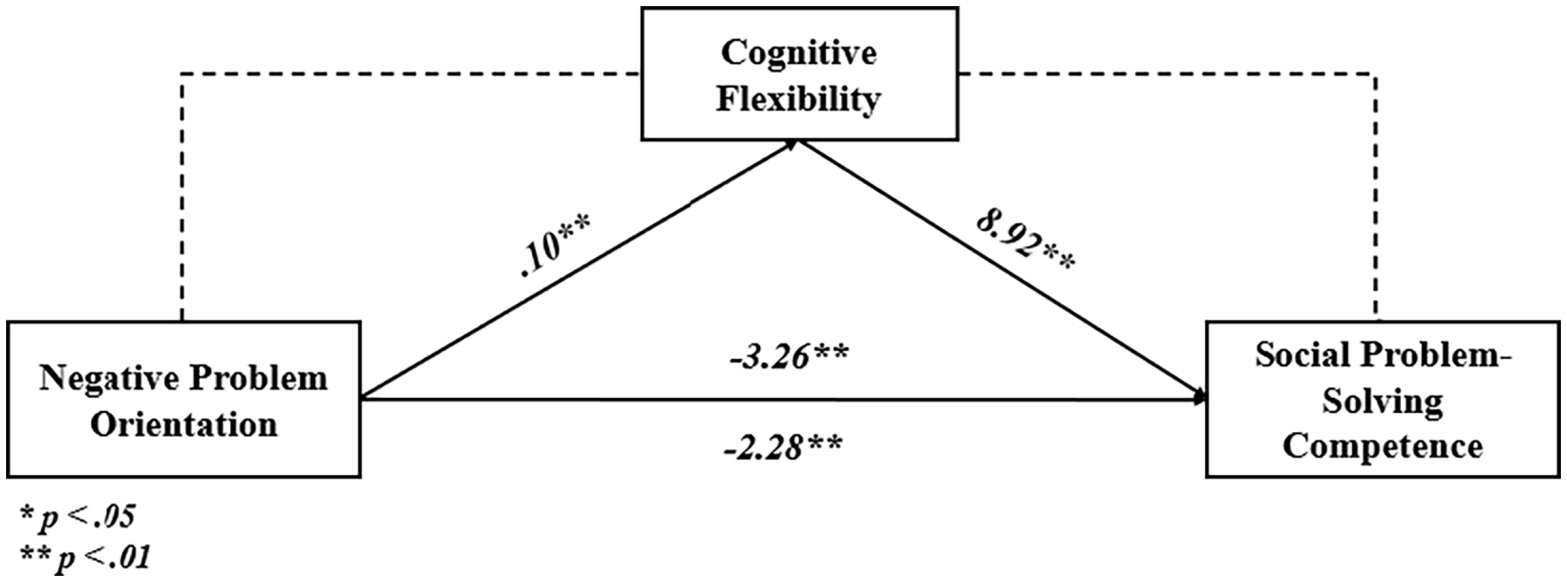
Cognitive flexibility mediates the relationship between negative problem orientation and social problem-solving competence.

The results regarding the mediating role of cognitive flexibility in the relationship between negative problem orientation and social problem-solving competence are shown in Table 3. The mediating role of cognitive flexibility in this relationship was analyzed using PROCESS Model 4 (Hayes, 2022; Bozkurt, 2023). The analysis was conducted with 5,000 bootstrap samples and a 95% confidence interval. The results indicated that cognitive flexibility has an indirect effect on the relationship between negative problem orientation and social problem-solving competence (Indirect Effect = 0.97; CI [0.4805–1.485]). Since the bootstrap confidence interval does not include zero, the mediating role of cognitive flexibility is significant. This demonstrates that the reducing of negative problem orientation on social problem-solving competence occurs through cognitive flexibility. The bootstrapping coefficient and 95% confidence intervals (CI) for the significance of the indirect effect related to mediation identified in the model in Figure 3 are presented in Table 4.

**Table 4:** Bootstrapping process of partial model.

Pathways	Effect	SE	*p*	LLCI	ULCI
Direct effects					
Negative Problem Orientation → Social Problem-Solving	−3.26	0.32	0.00	−3.894	−2.632
Total effects					
Negative Problem Orientation → Social Problem-Solving	−2.28	0.43	0.00	−3.134	−1.443
Indirect effect	Effect	BootSE		BootLLCI	BootULCI
Negative Problem Orientation → Cognitive Flexibility → Social Problem-Solving	0.97	0.25		0.4805	1.485

As shown in Table 4, the mediation model was found to be significant [*F* (2, 963) = 419.50, *p* < 0.001]. Bootstrapping analysis shown that cognitive flexibility significantly indirect roles the relationship between negative problem orientation and social problem-solving competence (Bootstrap coefficient = 0.97, 95% CI [0.4805, 1.485]). Specifically, higher negative problem orientation was correlations with lower cognitive flexibility, which in turn was associated with lower social problem-solving competence. Furthermore, as illustrated in Table 4, the direct effect from negative problem orientation to social problem-solving competence was significant and negative. The direct effect from cognitive flexibility to social problem-solving competence was also significant and positive. Together, these findings suggest a significant indirect effect; although negative problem orientation is generally associated with lower social problem-solving competence, it is also indirectly correlated with higher competence through increased cognitive flexibility.

## 4 Discussion

To understand the cognitive and emotional processes influencing individuals’ social problem-solving skills within the context of esports, this study identified three primary objectives: (a) to examine the relationship between negative problem orientation, social problem-solving skills, and cognitive flexibility; (b) to investigate the direct effect of negative problem orientation on social problem-solving skills; and (c) to determine whether cognitive flexibility mediates the relationship between negative problem orientation and social problem-solving skills.

Firstly, when examining the relationships between variables, it was determined that social problem-solving skills are positively related to cognitive flexibility and negatively related to negative problem orientation. This finding suggests that individuals who are more cognitively flexible tend to adopt more adaptive and constructive approaches to social problems. The robust association between cognitive flexibility and social problem-solving skills suggests that higher cognitive flexibility may facilitate the development of more effective solutions for social challenges. Effectively solving social problems requires the generation of multiple solutions (Crick and Dodge, 1994; Spivack et al., 1976). This relationship can be theoretically attributed to cognitive flexibility allowing for the generation of various alternatives and their evaluation in relation to environmental demands, thereby potentially yielding more adaptive responses (Anderson, 2002; Eslinger and Grattan, 1993).

The results obtained in this research are in concord with findings regarding the relationship between social problem-solving and cognitive flexibility. Sternheim et al. (2012) reported that cognitive flexibility impacts social problem-solving by providing an ability to adapt to feedback. Similarly, Buğa et al. (2018) found that university students with higher cognitive flexibility showed more effective problem-solving styles. Furthermore, Schäfer et al. (2024), Cañas et al. (2003), and Ionescu (2012) indicated that cognitive flexibility enhances problem-solving performance, while Hatashita-Wong et al. (2002) pointed out its mediating role in social problem-solving competence. In the context of esports, these cognitive parameters are considered relevant for adapting to the challenges of digital competitive environments. Although esports players are constantly engaged in social and cognitive processes such as team communication and opponent analysis, it is important to note that this study did not directly measure in-game performance indicators. However, it is theoretically plausible that players with higher cognitive flexibility might devise a greater number of strategies and adapt more effectively to sudden changes (Valls-Serrano et al., 2022). Effective social problem-solving may also potentially support team unity and ethical conduct. Nevertheless, further research is required to empirically validate whether these social competencies directly translate into enhanced competitive performance.

Negative problem orientation is known to adversely affect motivational ability and one’s belief in their ability to solve problems (D’Zurilla and Chang, 1995). This negative disposition can restrict the capability to handle interpersonal conflicts and communicate constructively. Our findings agree with literature indicating that individuals with a negative problem orientation view stressful situations as threatening, which impairs the development of appropriate coping strategies (Larson et al., 1990; MacNair and Elliott, 1992). In esports, where swift thinking and team decision-making are required, such an orientation could theoretically hinder the formulation of valid solutions during high-pressure moments. Therefore, decreasing negative problem orientation might be a significant factor in improving the broader problem-solving environment in competitive gaming.

Cognitive flexibility enables individuals to adapt their mental resources to environmental changes and modify behaviors in a goal-directed manner (Dennis and Vander Wal, 2010; Garcia-Garcia et al., 2010). Our results suggest that cognitive flexibility may mitigate the impact of negative problem orientation on social problem-solving skills by facilitating more innovative solutions (Conversano et al., 2010). This role is particularly relevant in fields like esports, where players must make dynamic decisions under shifting conditions. A negative problem orientation may lead players to view challenges as threats, which could potentially impair strategic thinking as seen in other high-stakes sports (Nagorsky and Wiemeyer, 2020). Enhancing the cognitive flexibility of esports players is therefore proposed as a potential strategy to overcome the deficits of negative problem orientation and support adaptability (Swettenham and Whitehead, 2022; Tang, 2018).

Finally, this study demonstrated that cognitive flexibility serves as a mediator, diminishing the weight of negative problem orientation and enhancing the effectiveness of problem-solving processes (Çelikkaleli and Gündüz B A, 2019; Rezapour Mirsaleh and Esmaeelbeigi Mahani, 2017). The findings imply that cognitively flexible players may maintain more functional attitudes in adverse conditions (Yarayan et al., 2023). While this study suggests that cognitive flexibility is a determinant in how players process social and situational problems, it does not provide direct evidence of improved win rates or specific game metrics. Future interventions focusing on these cognitive skills may offer a framework for supporting player development, provided that the link between social problem-solving and in-game success is further explored through empirical performance data.

## 5 Conclusion

The present study demonstrates that cognitive flexibility mediates the relationship between negative problem orientation and social problem-solving skills among esports players. Individuals with higher cognitive flexibility appear less vulnerable to the adverse effects of maladaptive problem orientations in terms of their social problem-solving competence.

These findings highlight the importance of flexible cognitive processing in regulating social-cognitive mechanisms within digitally mediated competitive contexts. Nevertheless, because the study did not assess objective esports performance outcomes, conclusions regarding performance enhancement or competitive advantage should not be drawn. Future research should directly investigate whether interventions targeting cognitive flexibility produce measurable improvements in in-game decision-making or competitive performance.

From a theoretical perspective, cognitive flexibility emerges as a central construct linking affective-cognitive appraisals and adaptive problem-solving processes. Further empirical validation within esports settings remains necessary to determine its applied implications.

## 6 Limitations

This study has several limitations, and its results should be interpreted with caution. First, the study utilized a cross-sectional, self-administered online survey for data collection, which may limit the generalizability of the findings. All measures used in the present study were self-report instruments, which may be considered a limitation due to potential response biases. Additionally, the sample consisted of individuals from Turkey categorized as Tier 2, Tier 3, professional, semi-professional, and hardcore players. However, the study did not provide specific information regarding which participants belonged to which category and game type (FPS, MOBA, MMPORG), which can be considered a significant limitation. The lack of categorization may have led to potential biases in the interpretation of results and hindered a more nuanced understanding of the differences among these groups.

## 7 Practical implications

Future studies could address this limitation by incorporating detailed information about participants’ professional levels. For instance, distinguishing between Tier 1 or solely professional players and other categories could provide deeper insights into the varying needs, cognitive flexibility, problem-solving skills, and negative problem orientation across different skill levels. Such stratification could enhance the applicability of research findings to specific players profiles and their unique psychological demands. Furthermore, considering that esports is often regarded as a team sport, future research could benefit from including scales that assess team-related dynamics, such as interpersonal communication, collaboration, and teamwork effectiveness. Incorporating such measures could provide a more comprehensive understanding of how cognitive flexibility and problem-solving skills interact within a team context, which is crucial for developing targeted interventions and training programs for esports players.

## Data Availability

The data supporting the findings of this study are available from the corresponding author upon request.
